# Reflections on the Intermediate Data Structure (IDS)

**DOI:** 10.51964/hlcs9570

**Published:** 2021-03-31

**Authors:** George Alter

**Affiliations:** University of Michigan

**Keywords:** Historical demography, Intermediate Data Structure, Data sharing, Life course, Metadata, Event history analysis

## Abstract

The Intermediate Data Structure (IDS) encourages sharing historical life course data by storing data in a common format. To encompass the complexity of life histories, IDS relies on data structures that are unfamiliar to most social scientists. This article examines four features of IDS that make it flexible and expandable: the Entity-Attribute-Value model, the relational database model, embedded metadata, and the Chronicle file. I also consider IDS from the perspective of current discussions about sharing data across scientific domains. We can find parallels to IDS in other fields that may lead to future innovations.

## 1 WHAT IS IDS?

The Intermediate Data Structure (IDS) emerged from a series of workshops held in Montreal (2003), Amsterdam (2006), and Ann Arbor (2008) to address the problem of sharing and re-using longitudinal historical data from family reconstitutions and population registers. The turning point in these meetings occurred in Amsterdam, when Kees Mandemakers proposed using the Entity-Attribute-Value model discussed below. Previous articles have described the problem that we were trying to solve (Alter, Mandemakers, & Gutmann, 2009) and refinements to the specification (Alter & Mandemakers, 2014). This article attempts to look at the IDS from a different perspective. I discuss four key aspects of IDS, including an important contribution by Luciana Quaranta, to consider what they do and why they are important.

As its name implies, IDS is a way station between the diverse data structures used for collecting data and the data format required for statistical analysis. Data used by historical demographers are derived from a variety of different sources, which vary from place to place and over time. In the end, all of this data must be transferred to a rectangular data array (‘dataframe’) compatible with standard statistical software. Moving data from one structure to another is a central problem in data science. Even though researchers in many domains make these transformations every day, everyone agrees that ‘data wrangling’ is the most cumbersome and time-consuming part of data-intensive research. The DDI Alliance recently released a new specification, the Data Documentation Initiative — Cross Domain Integration(DDI-CDI) model (DDI Alliance, 2020), to create a common language describing how data move from one format to another, which offers some context for IDS.

## 2 ENTITY-ATTRIBUTE-VALUE

The most radical difference between IDS and standard social science data is its use of an Entity-Attribute-Value (EAV) format. EAV explodes the standard rectangular data array by creating a new observation for every value in the data. Rows in an EAV table consist of a value (‘datum’ in DDI-CDI) describing an attribute (variable) about an entity (person, place, etc.).

**Figure 1:**
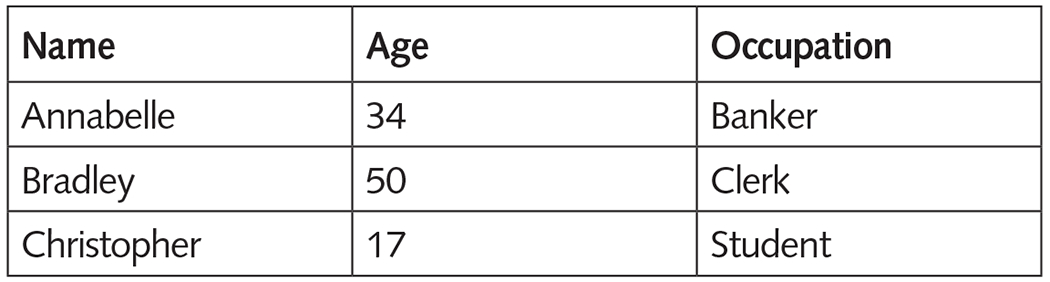
Wide Format Data

**Figure 2:**
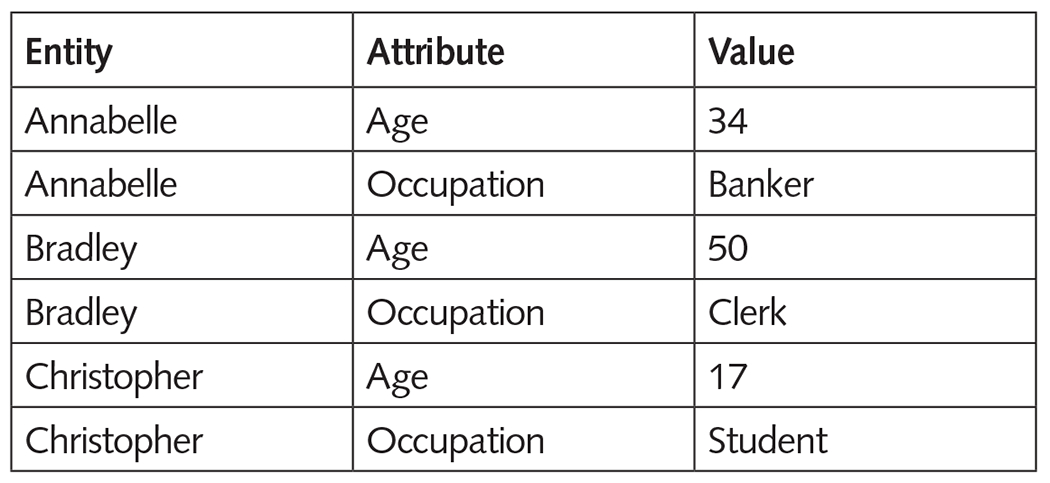
Entity-Attribute-Value Data Format

Figures 1 and 2 illustrate this difference. Each row in Figure 1 describes a person, and all of the variables pertaining to a person are on the same row. In Figure 2 there is a separate row for each attribute about each person. The data are all the same, but they are arranged in a different way. ‘Name’, which is a variable in Figure 1, is an identifier in Figure 2. ‘Age’ and ‘Occupation’, which were variable names (metadata) in Figure 1 become part of the data in Figure 2. Figure 1 is an example of what DDI-CDI calls ‘Wide’ data format, and Figure 2 is an example of ‘Long’ data format (DDI Alliance, 2020).

IDS has a number of similarities to the Observations & Measurements (O&M) standard (ISO 19156) developed for geographic information (Cox, 2011). Like IDS, O&M data are organized around ‘observed properties’ (attributes/types), and each observation can include a temporal attribute (timestamp). O&M observations may include indicators of data quality, like the Estimation and Missing fields in the IDS Timestamp. However, O&M and IDS have different ways of representing geography. Every observation in O&M can include spatial attributes, but IDS locates observations in space by creating relationships between individuals and contexts. The IDS approach is rooted in two important concerns in historical demography. First, the concept of ‘under observation’ or ‘at risk’ is central to demography. We need to know when a person was within the boundaries of the administrative unit (a context) that maintained surveillance of demographic events (births, deaths, marriages, migrations). Second, tracking relationships between individuals and contexts allows us to discover when two individuals were present in the same context (e.g., household) at the same time.

EAV has two advantages over other data structures for historical longitudinal data. First, it is a much more compact and efficient way of storing data on repeated events. For example, if we included columns for re-marriages in a ‘Wide’ format, we would create additional columns for each additional marriage, e.g. Marriage_date_1, Marriage_date_2, Marriage_date_3, etc. The number of columns would be determined by the person with the most marriages, which might be as high as five or six. Since most people only married once in the past, most of these columns would be empty.

Second, EAV is easily expandable. If a researcher discovers a new type of data, it can be added without any change to the structure of IDS. We expect all IDS databases to contain common demographic events (birth, death, marriage, migration) and personal attributes (sex, name, place of birth, occupation). But the historical sources used in longitudinal research vary widely, and they may include uncommon or unique variables, such as tax assessments, rents, heights, noble or honorary titles, religion, etc. IDS allows any database to add new data types to meet their needs.

## 3 THE RELATIONAL DATABASE MODEL

When IDS was designed, we expected that it would be used in a relational database. Three IDS tables are relational in an obvious way, because they define relationships among people and contexts. These tables can link related individuals across generations and find people living in the same household. Relational database systems operate on rectangular data arrays, called ‘tables’, but they perform very differently from statistical analysis software. Relational databases are optimized to retrieve specific items of information, and they are very good at combining (‘joining’) data from multiple tables to make new tables. These features make it easy to select all of the birth dates from the IDS INDIVIDUAL table or to link the birth dates of mother-child pairs found in the INDIV_INDIV table. Where the EAV aspect of IDS pulls data items apart, the relational model brings information together in a flexible and dynamic way.

The relational aspect of IDS affects how program code is written. EAV tables cannot be processed row by row, because each subject’s attributes are spread across many rows. Most computations on an IDS database involve linking rows from more than one table or multiple links to different rows in the same EAV table. Relational database languages, like SQL (Structured Query Language), are designed to link rows across tables, and R and Python include SQL-like commands for ‘joining’ dataframes. Achieving the same results with older languages, like SAS and Stata, requires ‘merging’ rows from different files.

## 4 METADATA

IDS was created to encourage sharing of both data and program scripts. Metadata is essential for data re-use, because researchers need to know what is being described and how it was measured. For example, the distinction between a birth date and a baptism date is important for some kinds of analyses. The Types used in an IDS database are included in the Metadata table with explanatory definitions. Values used in Types (e.g. single, married, divorced, etc. for marital status) are also included in the Metadata table. The IDS Metadata table encourages standardization, but individual databases have the flexibility to define local Types to describe unique and unusual data.

In the metadata world, the IDS Metadata table is a ‘controlled vocabulary’, and many disciplines have developed controlled vocabularies to remove ambiguity about the meaning of data. Ontologies are more advanced versions of controlled vocabularies that describe relationships among terms. Some fields, like biomedical research, rely heavily on controlled vocabularies and ontologies, and organizations exist to maintain and disseminate them. The Historical International Standard Classification of Occupations (HISCO) is an example of an ontology used in historical research. As IDS expands to include more databases, the Metadata table will become a reflection of how the field of historical demography is developing.

## 5 CHRONICLE FILE

The Chronicle file is a step in the transition from an IDS database to episode files for event history analysis introduced by Luciana Quaranta (2015). Event history analysis focuses on the timing of a specific event of interest, such as marriage, childbirth, or death. We want to know how various attributes of the subject are related to the probability that the event of interest will occur. Episode files divide life histories into segments of time defined by changes in the attributes of the subject or the environment. Attributes are constant within episodes, and a new episode begins when any attribute changes. Episode files for different events cover different parts of a life history. For example, only never-married people are at risk of first marriage, and only married people are at risk of widowhood or divorce. The event history ends when the subject experienced the event, became ineligible to experience the event, or when information about the subject ended. Quaranta’s work is important because it divides the process of creating an episode file into smaller, more manageable steps.

The Chronicle file is a list of dates when the value of a variable changed. Some of these dates can be taken directly from an IDS Type. For example, marital status is always ‘unmarried’ on BIRTH_DATE, changes to ‘married’ on MARRIAGE_DATE, and becomes ‘widowed’ on the DEATH_DATE of the spouse. Variables like ‘number of older co-resident unmarried brothers’ are extracted from IDS with more complicated programming.

The Chronicle file has two implications for creating episodes. First, if we select all unique dates from the Chronicle file, we have a list of the beginning and end dates of episodes. Second, if the value of a variable did not change at the beginning of an episode, we can copy its value from the previous episode. In other words, there is a straightforward path from the Chronicle file to the episode file.

Chronicle files are also useful because they are extensible and reusable. Since a row in a Chronicle file refers to only one variable, rows for each variable can be constructed separately and then combined. New variables can be added to an existing Chronicle file, and unnecessary variables can be dropped. Similarly, we can easily repurpose a Chronicle file for a different analysis. A Chronicle file for mortality can be reused to study migration with minimal changes.

## 6 CONCLUSION

The problems that IDS was designed to solve are not simple, and they are not unique to historical demography. Life histories unfold in many ways, and IDS is a tool for capturing dynamic relationships between individuals like kinship and coresidence. IDS was also designed with flexibility and expandability to capture the variety of data types found in historical sources. Flexibility has costs because complexity makes writing computer programs more difficult, but these costs can be offset by sharing and reusing those programs. As interdisciplinary discussions about data sharing and data interoperability increase, we can see that IDS shares important features with solutions used in other scientific domains. Situating IDS in the broader data ecosystem will make it possible to learn from other disciplines.
